# What do I do to avoid sexually transmitted diseases? A scale development study: behavioral scale for protection from sexually transmitted diseases

**DOI:** 10.1186/s12905-023-02658-9

**Published:** 2023-09-21

**Authors:** Mustafa Kılavuz, Feride Yigit

**Affiliations:** 1 Department of Midwifery, Istınye University, Istanbul, Türkiye; 2 Faculty of Health Sciences, Topkapi University, Istanbul, Turkey

**Keywords:** Sexually transmitted diseases, Scale development, Validity, Reliability, Women health

## Abstract

The authors’ aim is to develop and confirm a reliable and valid measurement tool that can measure women’s behavior to prevent sexually transmitted diseases. The study data were collected from adult women aged 18–47 years (n = 505). Explanatory factor analysis (EFA) was applied to the STD scale consisting of 44 items in a 5-point Likert style. According to the results of EFA, 21 items were defined in the scale. The KMO value of the scale is 90.6%. In the study, the varimax rotation method was used to bring the factors together with the related items. As a result of varimax rotation, two factors with eigenvalues greater than 1 were found in the scale. The overall Cronbach’s alpha coefficient of the scale was calculated as 0.911, 0.941 for the first subdimension and 0.889 for the second subdimension. The validity and reliability of this study have been proven according to the results of explanatory factor analysis (EFA) and confirmatory factor analysis (CFA).

## Background

The term “sexually transmitted infections” (STIs) refers to a pathogen that causes infection through sexual contact, while the term “sexually transmitted disease” (STD) refers to a disease caused by viruses, bacteria, fungi, parasites, protozoa or arthropods that are usually transmitted through sexual contact [1, 2]. Reported disease rates underestimate the true burden of infection because the majority of STDs are asymptomatic and due to underreporting [3]. From the Centers for Disease Control and Prevention (CDC) STD surveillance in 2020, it is obvious that STDs remain a major public health concern, even in the face of a pandemic [4].

More than 1 million sexually transmitted infections (STIs) are acquired every day worldwide, the majority of which are asymptomatic. Each year there are an estimated 374 million new infections with 1 of 4 curable STIs: chlamydia, gonorrhoea, syphilis and trichomoniasis [5]. It is estimated that 46.8 million of 450 million people in the 15–49 age group living in the European region, including Turkey, have a treatable STD [6].

Undiagnosed STIs can cause significant health complications that put women at risk for pelvic inflammatory disease, ectopic pregnancy, and tubal factor infertility [7]. Indeed, because STDs are communicable diseases with far-reaching public health consequences, early detection and treatment are important; therefore, clinicians can play a role not only in improving the sexual and reproductive health of individual patients but also in improving the long-term health and health care costs of their communities [8].

In the literature, sexually transmitted diseases mostly consist of concepts related to the level of knowledge, awareness or any prevention method [9–11]. It has been observed that the studies conducted to measure the protection behaviors from sexually transmitted diseases were carried out without relying on a measurement tool. Therefore, it is believed that this scale makes a significant contribution to the literature and has original value. Developing and validating a scale for protection from sexually transmitted diseases will first provide researchers with analysis results on women’s behavior. The research will be able to determine the programs for healthy prevention behaviors by identifying the positive and negative factors related to the subject. In the literature review, no valid and reliable measurement tool was found to be used to evaluate the status of women regarding protection from sexually transmitted diseases. The aim of the authors in this study is to develop a scale whose validity and reliability are confirmed and that can measure women’s behaviors to protect against sexually transmitted diseases. For this reason, this scale will make a significant contribution to the literature and has a unique value.

## Methods

### Design

It is a methodologically designed scale development study.

### Stage 1: preparation

First, we developed a pool of items based on a literature review. From the perspective of protecting against sexually transmitted diseases, we searched PubMed, Web of Science, Tr Index, and Google-web for related articles. We used specific key words as follows: protect from sexually, nursing role for protect from sexually transmitted diseases, signs and symptoms of STDs. transmitted diseases [12–15].

An item pool of 60 questions was created by scanning the literature by the researchers. Ten professionals provided support to solve the spelling and expression problems of the created item pool. In order to evaluate the validity of the scope, 9 academic members specialized in the field of item pool, Obstetrics and Gynecology Nursing were consulted. Expert opinions were evaluated by the Davis technique conducted by the researchers. Sixteen items in the scale were below the low content validity index (CVI) value, and the CVI value of the other 44 items was found to be higher than 0.80. To evaluate the test-retest reliability, the scale was reapplied to 50 participants after a certain time. The consistency between the first and last test results obtained from the same 50 participants was evaluated with the intraclass correlation coefficient (ICC), and the significant differences within the group were evaluated with the Wilcoxon test. As a result of the test performed for the first and second subdimension scores of the scale, the ICC coefficient was found to be 1 and 0.945, respectively, and it was found to be statistically significant and highly compatible. A five-point Likert-type scoring was used to express the levels of agreement with the items in the draft scale. This scoring included options such as “Absolutely Disagree, Disagree, Moderately Agree, Agree, and Absolutely Agree.” The maximum score obtained from the scale with 21 items will be 105, and the minimum score will be 21.

### Stage 2: sampling and permissions

Participants between the ages of 18–49, who had not had a sexually transmitted disease before and who were able to read and understand the questions took place in the study.

The study permit was obtained from the Non-Interventional Ethics Committee of the Faculty of Health Sciences, Hasan Kalyoncu University, in 2021. (Decision no: 2021/087). Research ethics principles for consent, information and confidentiality according to the Declaration of Helsinki were taken into account during the work on the study. Written and verbal consent was obtained from the participants before the data were collected, and all information remained confidential.

Data were collected through questionnaires from women aged 18–49 between January 2022 and February 2022. According to the number of sample items, at least 440 female participants were required in the study. In total, 505 women participated in the study [16].

### Stage 3: implementation

#### Data collection

The data were collected using the demographic data form created by the researchers through a literature review and the developed behavioral scale for protection from sexually transmitted diseases for women. The demographic data form consists of 13 questions, and these questions were asked about the descriptive characteristics of women and sexually transmitted diseases. The behavior scale for protection from sexually transmitted diseases for women consisted of 21 questions.

#### Data analysis

The “Behavior Scale for Protection from Sexually Transmitted Diseases (STD scale)”, which was planned to be developed in this study, was applied to 505 participants. For the validity and reliability analysis of the scale, descriptive statistics were calculated for each item score and scale score. To determine the reliability of the scale, correlation-based item analysis was applied. The internal consistency reliability of the scale was examined with Cronbach’s alpha coefficient, which determines the similarity level of the items and the differentiation of the structure that the items want to measure, and the item-total correlation, which determines the adequacy of the scale items in distinguishing individuals in terms of the scale. It can be said that items with a total score correlation of 0.30 and higher distinguish individuals well. In addition, in determining the reliability of the scale, the test-retest reliability of the answers given by re-administering the “STD” scale to the same 50 participants intermittently was examined. Explanatory factor analysis (EFA) was used for the validity of the scale, and confirmatory factor analysis (DFA) was performed for the construct validity of the scale. Descriptive statistics of numerical data were expressed as the mean ± standard deviation (min-max), and descriptive statistics of categorical data were expressed as frequency (percent). EFA and descriptive statistical analyses were performed using IBM SPSS Statistics 26.0, and DFA analysis was performed using the LISREL 8.7 program. All analyses were examined at the 95% confidence level, α = 0.05 significance level.

## Results

A total of 505 participants participated in the study. The mean age of the participants was 23.82 ± 5.74 (min: 18, max: 47). When the education level of the participants was examined, 1 (0%) participant had a primary school education, 33 (6.5%) participants had a secondary school education, 171 (33.9%) participants had a high school education, 262 (51.9%) participants had an associate’s degree, and 38 (7.5%) participants had a graduate education. When the income distribution is examined, it is found that the income of 228 (45.1%) participants is less than their expenditure, the income of 210 (41.6%) participants is equal to their expenditure, and the income of 67 (13.3%) participants is more than their expenditure. A total of 413 (81.8%) of the participants did not have any addiction, 6 (1.2%) were addicted to alcohol, and 86 (17%) were addicted to cigarettes. A total of 171 (33.9%) of the participants had a relationship, and 334 (66.1%) had no relationship. The number of participants with an active sexual life was 74 (14.7%). A total of 436 (86.3%) of the participants had knowledge about STDs. (Table 1.)

**Table 1:** Participant characteristics

Demographic Data	Descriptive Statistics		
	mean ± standard deviation(min:max)		
Age	23.82 ± 5.74 (min: 18. max:47)		
	n	**%**	
Education Level			
Primary school	1	0.20%	
Middle School	33	6.50%	
High school	171	33.90%	
Associate-Bachelor’s	262	51.90%	
Graduate	38	7.50%	
Income			
Income less than expenditures	228	45.10%	
Income equals to expenditures	210	41.60%	
Income more than expenditures	67	13.30%	
Addiction			
Alcohol	6	1.20%	
Cigarette	86	17.00%	
No	413	81.80%	
Relationship Status			
I’m in a Relationship	171	33.90%	
I have no relationship	334	66.10%	
Do you have an active sex life?			
Yes	74	14.70%	
No	431	85.30%	
Do you have any information about STD?			
Yes	436	86.30%	
No	69	13.70%	
What is Information Source			
Magazine. Book. Newspaper	102	20.20%	
School	195	38.60%	
Internet. TV	316	62.60%	
Family. friend	112	22.20%	
Health Institution	147	29.10%	
Which STDs do you know?			
AIDS	419	83.00%	
Syphilis	208	41.20%	
chlamydia	248	49.10%	
hepatitis B	249	49.30%	
Trichomonas	349	69.10%	
hepatitis C	175	34.70%	
Herpes (herpes simplex)	131	25.90%	
HPV	244	48.30%	
Gonorrhea	214	42.40%	
What do you know about STD symptoms?			
redness	219	43.40%	
Itching	263	52.10%	
Vaginal discharge	241	47.70%	
Pain during sexual intercourse	329	65.10%	
Fever	176	34.90%	
Skin eruption	302	59.80%	
Weakness	188	37.20%	
Weight loss	160	31.70%	
burning while urinating	245	48.50%	
diarrhea, nausea	180	35.60%	
Can sexually transmitted diseases be treated?			
Yes	309	61.20%	
No	44	8.70%	
I do not know	152	30.10%	
Are common areas effective in STD transmission?			
Yes	303	60.00%	
No	137	27.10%	
I do not know	65	12.90%	
Does having an STD affect your psychology negatively?			
Yes	489	96.80%	
No	***16***	***3.20%***	

*Descriptive statistics of data are expressed as frequency (percentage) and mean ± standard deviation

According to Table 2, the KMO value is 90.6%. A KMO test result greater than 60% and a significant Bartlett test result (p < 0.001) indicate that the scale is suitable for factor analysis. Therefore, there are correlations between the items.

**Table 2:** Test Results for Factor Analysis Suitability

Kaiser-Meyer-Olkin Measure of Sampling Adequacy	0.906
Bartlett’s Test of Sphericity	p-value <0.001

In the study, the varimax rotation method was used to bring the factors together with the related items. As a result of Varimax rotation, two factors with eigenvalues ​​greater than 1 were found in the scale: (1) Factor eigenvalue = 8.595, and (2) Factor eigenvalue = 4.146. The percentages of variance explained by both factors are 38.161 and 19.753, respectively. The total variance explained is 57.914%. Thus, it is concluded that the STD scale is two-dimensional. The factor loads of the two-factor STD behavior scale, which was created as a result of the EFA applied, and the percentage of total variance they explained are summarized in Table 3.

**Table 3:** Explanatory Factor Analysis Information of the STD Behavior Scale

			Varimax Rotated Factor Loads	
Scale Items	*I*	*M*	*SD*	
**Factor 1**				
If I suspect a sexually transmitted disease, I go to a health institution	0.776	4.202	1.166	
I use a condom (sheath) to protect myself from sexually transmitted infection.	0.807	3.848	1.24	
I have unprotected intercourse with someone who has a sexually transmitted disease unless the disease is fully treated	0.735	3.947	1.21	
I track whether I have symptoms of sexually transmitted diseases	**0.737**	3.8	1.311	
I get the necessary vaccinations to protect against sexually transmitted diseases	**0.826**	3.79	1.202	
I do not go to the doctor if I do not have symptoms of a sexually transmitted disease	**0.796**	3.733	1.222	
If I have a sexual illness, I do not tell my partner (spouse, lover, dating, etc.).	**0.755**	3.8	1.313	
Since sexually transmitted diseases can be transmitted without symptoms, I definitely take precautions.	**0.706**	3.727	1.22	
I ask my partner (spouse, lover, dating, etc.) to use an unused condom (sheath) in every sexual intercourse.	**0.748**	4.172	1.228	
In sexually transmitted diseases, only the sick partner should be treated.	**0.816**	3.953	1.129	
I participate in early diagnosis and screening programs to prevent sexually transmitted diseases.	**0.727**	3.867	1.153	
I share items with someone who has an STD	**0.714**	4.127	1.207	
If I am not sexually active, I will not get a sexually transmitted disease.	**0.667**	3.826	1.293	
Sexually transmitted diseases are transmitted only to individuals of the same sex.	**0.683**	3.335	1.254	
**Factor 2**				
I regularly track changes in my vaginal discharge.	**0.719**	4,093	1,095	
If I bleed after sexual intercourse, I immediately go to the doctor.	**0.862**	4,034	1,118	
In order not to have a sexually transmitted disease, I wash my vagina with water and vinegar after sexual intercourse.	**0.721**	3,556	1,251	
Sex during menstruation increases the transmission risk of a sexually transmitted disease.	**0.818**	4,143	1,063	
I hesitate to be in the same environment with someone who has a sexually transmitted disease.	**0.806**	3,984	1,188	
If I suspect a sexually transmitted disease, I first try to get information from social media.	**0.728**	4,016	1,061	
When I have pain during sexual intercourse, I suspect that I have a sexually transmitted disease.	**0.76**	3,992	1,195	
**Ratio of Variance Explained**			**57.91%**	

M: mean, SD: standard deviation I: vector loads

When Table 3 is examined, it can be said that the scale can explain attitudes towards STDs well. According to factor analysis, two subdimensions of the scale are shown in Table 3. Finally, the internal consistency coefficient of the scale consisting of 21 items was calculated. The overall Cronbach’s alpha coefficient of the scale was calculated as 0.911 for the first sub-dimension, 0.941 for the second sub-dimension, and 0.889 for the second sub-dimension. The results regarding the reliability coefficient are given in Table 4.

**Table 4:** Cronbach’s alpha coefficient and interitem difference results for the STD Behavior Scale

Scale	Number of Items	Cronbach Alpha		Hotelling T2 test p-value
		Raw alpha	Standardized Alpha	
Total	21	0.911	0.910	**p < 0.001**
Factor 1	14	0.941	0.941	**p < 0.001**
Fakctor 2	7	0.889	0.890	**p < 0.001**

When Table 4 was examined, it was concluded that the internal consistency of the scale was sufficient, and the difference between the scale items was statistically significant according to the Hotelling T2 test (p < 0.001).

To evaluate the test-retest reliability of the developed scale, the scale was reapplied to 50 participants after a certain time. The consistency between the first and last test results obtained from the same 50 participants was evaluated with the intraclass correlation coefficient (ICC), and the significant differences within the group were evaluated with the Wilcoxon test. The results are given in Table 5.

**Table 5:** Test-Retest Reliability Results

Test-Retest	Median(min-max)	p-value^a^	ICC	p-value^b^
Total Score				
Test	91(26–105)	0.067	0.656	**p < 0.001**
Re-test	76(26–96)			
Sub-dimension 1				
Test	63(14–70)	0.655	1	**p < 0.001**
Re-test	63.50(17–70)			
Sub-dimension 2				
Test	30.50(10–35)	0.596	0.945	**p < 0.001**
Re-test	28(7–32)			

a: p-value is the p-value of the Wilcoxon test. b: The p value of the ICC analysis. Data are expressed as the median (min-max)

When Table 5 is examined, the ICC coefficient of the test-retest results for the total score of the scale indicates that there is statistically significant and moderate compatibility. The difference between the means of test-retest results, on the other hand, does not have a statistically significant difference. As a result of the test-retest performed for the first and second subdimension scores of the scale, the ICC coefficient was found to be 1 and 0.945, respectively, and it was found to be statistically significant and highly compatible. In addition, the difference between the averages of the test-retest results of both the first and second subdimension scores is not statistically significant. In light of these results, the test-retest reliability of the STD Behavior Scale is also ensured.

Confirmatory factor analysis (CFA) was applied to examine the two-factor construct validity of the STD Behavior scale created by EFA. The model to be tested in line with examining the construct validity of the EFA results was created using 21 observed and two latent variables (Factor 1 and Factor 2). Analyses were made using the LISREL 8.7 program. The fit index values for the measurement model were found to be ${\chi }^{2}/SD$ =1.39 (p < 0.001), CFI = 0.97, RMSEA = 0.04, NNFI=0.96, NFI=0.89, SRMR=0.07. As a result of CFA, the fit index values indicate that the model and the data are compatible. The measurement model and the goodness of fit criteria for the model are given in Table 6.

**Table 6:** Goodness of Fit Indexes

Goodness of Fit Indexes	Correspondence Indicator	Result
Ki-Kare/SD	≤ 4–5	1.39
RMSEA	0 ≤ RMSEA ≤ 0.08	0.04
CFI	0.90 ≤ CFI ≤ 1	0.97
NNFI	0.90 ≤ NNFI ≤ 1	0.96
NFI	0.90 ≤ NFI ≤ 1	0.89
SRMR	0 ≤ SRMR ≤ 0.08	0.07

## Discussion

The aim of the researchers was to develop a valid and reliable measurement tool to evaluate their behaviour towards protection from sexually transmitted diseases. As far as is known, there is no standard measurement tool on this subject, although studies have been carried out to protect against sexually transmitted diseases in Turkey. The researchers of the studies conducted mostly examine the knowledge levels and attitudes of the participants about sexually transmitted diseases [10, 15, 17, 18]. Evaluation of the work done by professional experts is limited. For this reason, a behavioral scale for the prevention of sexually transmitted diseases was designed. To the best of our knowledge, it is the first measurement tool developed in this field. As the first step of scale development, it is stated that the concepts suitable for the content and purpose of the theoretical concept should be selected, conceptual definitions should be made, items and subscales should be formed, and feedback and expert opinion should be sought [19]. The suggested steps were followed in the scale development phase of the research.

Content validity tests whether the scale covers all relevant items necessary to answer the research question [20, 21]. The item pool, which was created by scanning the literature, was submitted to the evaluation of 9 experts who are experts in their field in order to determine the content validity. These views were evaluated by Davis method. According to Davis method, to determine whether the items are statistically significant; The CVR of each item in the scale and the total CGI of the scale were calculated. In order for the content validity to be sufficient, the content validity index must be above “0.80” [22]. According to the Davis Method, items below the value of 0.80 were excluded from the scale. After content validity, 44 items remained in the scale. While the CVR values of the items were found to be between 0.44 and 1, the total CGI value of the scale was found to be 0.89. According to the results, it can be said that the content/content validity of the Scale of Behaviors for Protection from Sexually Transmitted Diseases is ensured and the representation power is high in the area it aims to measure. The fact that the KMO value is greater than 0.60 and the result of Bartlett’s sphericity test is less than p < 0.05 shows that the tests are meaningful, and the data obtained are suitable for factor analysis [23]. In the research conducted by Yılmaz and Karahan (2019), the KMO value was 0.81, and the result obtained from Bartlett’s sphericity test was found to be significant [24]. In another scale development study, the KMO value was 0.917, and as a result of the Bartlett sphericity test, it was concluded that the data were suitable for factor analysis [25]. According to the results of this research, the KMO value was 90.6%, and Bartlett’s sphericity result was p < 0.001.

The total variance rate of the scale development items made by Başkaya et al. (2020) was 63.84%, and the Cronbach alpha reliability coefficient was 0.905 [26]. In the study conducted by Karakoç and Özerdoğan (2022), the variance rate of the scale was 47.13% for the first factor and 49.12% for the second factor [19]. According to the results of the research, the total variance rate of the scale was found to be 57.91%, and it was seen that it took place under two factors. The overall Cronbach’s alpha coefficient of the scale was calculated as 0.911, 0.941 for the first sub-dimension and 0.889 for the second sub-dimension.

### Limitations

The results of the study can be generalized to women between the ages of 18–49 constituting the sample group. The developed scale can be applied to women between the ages of 18–49. Since the behavioral scale for protection against sexually transmitted diseases was prepared in Turkish, some studies should be carried out to determine its validity and reliability in other cultures.

## Conclusion

A valid and reliable 5-point Likert-type Behavior Scale for Protection from Sexually Transmitted Diseases consists of 2 factors and 21 questions.

## Data Availability

All relevant data are found within the paper.
